# Discharge readiness and associated factors among thyroid cancer patients under ERAS-based perioperative management: a cross-sectional study

**DOI:** 10.3389/fpsyg.2026.1954989

**Published:** 2026-10-26

**Authors:** Yajun Wang, Shanshan Li, Lei Guo, Junyi Gao, Xiaotian Zhang, Danping Song, Bei Yang, Xiangyun Yin, Weinan Liu

**Affiliations:** 1 Department of General Surgery, Peking Union Medical College Hospital, Chinese Academy of Medical Sciences & Peking Union Medical College, Beijing, China; 2 Department of Thoracic Surgery, Peking Union Medical College Hospital, Chinese Academy of Medical Sciences & Peking Union Medical College, Beijing, China

**Keywords:** anxiety and depression, discharge readiness, Enhanced Recovery After Surgery, social support, thyroid cancer

## Abstract

**Objective:**

To investigate the current status of discharge readiness and its associated factors among postoperative thyroid cancer patients receiving ERAS-based perioperative management.

**Methods:**

A convenience sampling method was employed to select 154 discharged patients with thyroid cancer who underwent surgery under the ERAS protocol from the department of general surgery at Peking Union Medical College Hospital as the research objects. They were investigated with a general information questionnaires, Readiness for Hospital Discharge Scale (RHDS), Hospital Anxiety and Depression Scale (HADS), and Social Support Rating Scale (SSRS). Pearson correlation analysis was used to analyze the correlation between readiness for hospital discharge, hospital anxiety and depression, and social support levels for patients. Univariate analysis was used to screen independent variables based on literature support, and multiple stepwise regression analysis was employed to identify the factors associated with patients’ RHDS.

**Results:**

The total score of RHDS in patients with thyroid cancer after surgery was 95.25 ± 13.21 (mean item score: 7.94 ± 1.10). The total RHDS score was negatively correlated with the total HADS score (*r* = −0.777, *P* < 0.01), and positively correlated with the total SSRS score (*r* = 0.729, *P* < 0.01). Multiple linear regression analysis showed that the total score of HADS (*B* = −1.077, 95% CI: −1.424 to −0.731) and SSRS (*B* = 0.472, 95% CI: 0.155–0.789) were significantly associated with RHDS scores (*P* < 0.05). The final regression model explained 62.6% of the variance in RHDS scores (*R*^2^ = 0.626, adjusted *R*^2^ = 0.621).

**Conclusion:**

Among postoperative thyroid cancer patients receiving ERAS-based perioperative management, psychological distress and social support were associated with discharge readiness. These findings highlight the importance of incorporating psychological assessment, social support evaluation, and individualized discharge preparation into perioperative care. Tailoring discharge planning to patients’ individual needs and involving family caregivers when appropriate may help facilitate the transition from hospital to home.

## 1 Introduction

Thyroid oncology is a malignant neoplasm originating from thyroid follicular epithelial or parafollicular cells (Li et al., 2025; National Health Commission of the People’s Republic of China Medical Administration and Hospital Administration, 2022). In recent years, its incidence has increased rapidly worldwide. Epidemiological studies indicate that thyroid cancer ranks as the 9th most common malignancy globally, and in China, it holds the 3rd position in terms of newly diagnosed cancer cases (Han et al., 2024). Currently, surgery remains the primary therapeutic approach for thyroid cancer. With the growing adoption of the Enhanced Recovery After Surgery (ERAS) protocol in clinical settings, there has been a shift toward multidisciplinary collaboration to optimize perioperative clinical pathways. This approach aims to reduce patient stress and postoperative complications, shorten hospital stays, and accelerate recovery, benefiting an increasing number of patients (Althans et al., 2024; Chinese Society of Surgery, Chinese Medical Association, Chinese Society of Anesthesiology, Chinese Medical Association, 2021; Jain et al., 2023; Kehlet, 2022). However, as the length of hospital stay is shortened, the issue of whether patients are adequately prepared for discharge has not received sufficient attention. Readiness for hospital discharge refers to the comprehensive assessment by healthcare professionals of a patient’s physiological, psychological, and social health status, analyzing and determining whether the patient has the ability to leave the hospital, reintegrate into society, and achieve further recovery (Feldbusch et al., 2024; Galvin et al., 2017; Hreńczuk et al., 2025; Lu et al., 2021). A higher level of discharge readiness can reduce the rate of unplanned readmissions, promote postoperative recovery and clinical outcomes, while facilitating the effective integration of medical resources, thereby maximizing the utilization efficiency of healthcare resources (China Gerontological Nursing Alliance, 2020; Homem et al., 2024; Mu et al., 2025). Although the ERAS protocol has been shown to further shorten postoperative hospital stays and promote recovery in patients, limited evidence has specifically examined discharge readiness among thyroid cancer patients managed within an ERAS pathway. Previous studies have shown that psychological distress may be associated with lower discharge readiness (Lu et al., 2021), whereas greater social support may facilitate patients’ confidence in self-management and recovery after discharge (Presciutti et al., 2024; Rattanakanlaya et al., 2023). However, the relationships between psychological distress, social support, and discharge readiness in thyroid cancer patients remain insufficiently characterized. Therefore, this study aims to investigate the current status and associated factors of discharge readiness among these patients under the ERAS framework. The findings are expected to provide evidence to inform discharge preparation strategies for postoperative thyroid cancer patients receiving ERAS-based perioperative care.

## 2 Materials and methods

### 2.1 Study subjects

This is a cross-sectional study and approved by the ethics committee of Peking Union Medical College Hospital. From December 2025 to July 2026, we selected 154 patients with thyroid cancer and all patients were managed under the Enhanced Recovery After Surgery (ERAS) concept in the Department of General Surgery at our hospital. In this study, ERAS served as the clinical care context rather than an exposure or intervention for comparison. Accordingly, the study was designed to investigate readiness for hospital discharge and its associated factors among patients managed under an ERAS pathway, rather than to evaluate the effectiveness of ERAS.

The ERAS protocol implemented for thyroid cancer surgery included standardized preoperative education, perioperative optimization, early postoperative mobilization, early oral intake as clinically appropriate, multimodal pain management, and standardized discharge education before discharge. Discharge decisions were made by the treating surgical team according to routine clinical practice, including stable vital signs, satisfactory wound condition, adequate pain control, independent oral intake, and the ability to perform basic self-care at home.

Sample size calculation: the sample size was determined according to Green’s rule of thumb for multiple linear regression (Green, 1991). With 10 candidate predictors (age, gender, education level, marital status, monthly household per capita income, length of hospital stay, living status, and presence of similar cases among relatives or friends, the total HADS score, and the total SSRS score) predefined for entry into the multivariable regression model, the formula N ≥ 50 + 8 m (m = number of predictor variables) indicated that at least 130 participants were needed to test the overall regression model. This rule assumes a medium effect size, α = 0.05, and statistical power of 0.80. Our final analytical sample comprised 154 participants, which surpassed this threshold and ensuring adequate statistical power for the planned regression analysis.

Inclusion Criteria: over 18 years old; pathologically confirmed thyroid cancer and had undergone surgical treatment; Adopted the ERAS perioperative management protocol; no other severe diseases or complications (such as liver or kidney failure, distant metastasis of malignant tumors, coagulation dysfunction, etc.); Informed consent: patients or their legal guardians must signed an informed consent form agreeing to participate in this study and complete the relevant questionnaires.

Exclusion Criteria: presence of mental or psychiatric disorders preventing completion of the questionnaire; language or literacy barriers that would hinder comprehension or completion of the questionnaire.

### 2.2 Methods

#### 2.2.1 The general information questionnaire

This included 10 items covering patients’ age, gender, educational level, occupation, marital status, living status, medical payment method, monthly household per capita income, presence of similar cases among relatives or friends, and length of hospital stay.

#### 2.2.2 Readiness for Hospital Discharge Scale (RHDS)

Developed by Weiss and Piacentine (2006) and translated into Chinese by Lin et al. (2014), the RHDS consists of 12 items across three dimensions: personal status, coping ability, and expected support. Each item is scored from 0 to 10, with a total score ranging from 0 to 120; higher scores indicate better readiness for discharge. The Cronbach’s α for the total scale was 0.89, with dimension-specific values of 0.73, 0.90, and 0.89.

#### 2.2.3 Hospital Anxiety and Depression Scale (HADS)

Developed by Zigmond and Snaith (1983), the HADS is widely used to screen for anxiety and depression in general hospital patients. It comprises 14 items, with two subscales for anxiety and depression, each containing seven alternating items. Higher scores indicate more severe anxiety or depressive symptoms.

#### 2.2.4 Social Support Rating Scale (SSRS)

Designed by Xiao (1994), the SSRS consists of 10 items across three dimensions: objective support, subjective support, and utilization of social support. Total scores are obtained by summing all item scores, with higher scores indicating better social support. The scale demonstrated good reliability and validity, with a Cronbach’s α of 0.896.

### 2.3 Data collection and quality control

This study distributed paper-based anonymous questionnaires within 4 h on the day patients were confirmed discharged. Once the attending physician confirmed the discharge decision, standardized discharge education was provided by the healthcare staff, following which the patients independently completed the questionnaire to ensure data reliability and validity. Questionnaires were collected on-site immediately after completion, and any missing items were promptly supplemented. The entire process took approximately 20 min.

Data entry personnel were trained on the system’s usage, and all data were independently entered by two researchers who then conducted manual verification and discrepancy comparison, promptly correcting any errors or omissions. A total of 162 questionnaires were distributed, with 154 valid responses received, yielding an effective response rate of 95.1%.

### 2.4 Statistical analysis

All data were analyzed using SPSS 26.0 statistical software. Categorical variables were presented as frequencies and percentages, and continuous variables were expressed as mean ± standard deviation (SD). Univariate analyses were performed using *t*-tests or one-way ANOVA. Pearson correlation analysis was conducted to assess the relationships between patients’ readiness for hospital discharge and hospital anxiety and depression scores as well as social support ratings.

Candidate predictors were chosen based on clinical relevance, evidence from previous literature, and the results of univariate analyses. Clinically relevant risk factors recognized in the existing literature, together with variables with *P* < 0.05 in the univariate analyses, were considered for inclusion in the multivariable model. Age and gender were retained as conventional demographic confounders regardless of their univariate significance. A stepwise multiple linear regression approach was adopted because the study was exploratory in nature and aimed to identify variables independently associated with RHDS scores while developing a parsimonious model. Before building the final regression model, multicollinearity was checked using variance inflation factor (VIF). Residual plots were inspected to verify linearity and homoscedasticity, and the distribution of standardized residuals was checked for normality. Cook’s distance was calculated to identify influential observations in the multiple linear regression model.

## 3 Results

### 3.1 Comparison of RHDS scores among postoperative thyroid cancer patients with different demographic characteristics

The results of this study indicated that, among the 10 demographic characteristics surveyed, the difference in RHDS scores between male and female patients was statistically significant (*P* < 0.05). The differences in the remaining nine characteristics were not statistically significant (*P* > 0.05). Detailed results are presented in Table 1.

**TABLE 1:** Comparison of RHDS scores among postoperative thyroid cancer patients with different demographic characteristics (*N* = 154).

Variable	Total *N* = 154	RHDS scores (x̄ ± s)	*t/F*	*P*
Age (years)			0.637	0.530
18∼44	95 (61.7%)	95.15 ± 12.85		
45∼59	48 (31.2%)	96.33 ± 14.50		
≥60	11 (7.1%)	91.36 ± 10.33		
Gender			−2.236	0.027
Male	41 (26.6%)	99.15 ± 11.70		
Female	113 (73.4%)	93.83 ± 13.49		
Education level			1.783	0.153
Bachelor’s degree or higher	93 (60.4%)	96.26 ± 12.94		
College diploma*	29 (18.8%)	94.83 ± 11.51		
High school education	17 (11.0%)	88.59 ± 15.53		
Junior high school education or below	15 (9.7%)	97.33 ± 14.14		
Occupation			1.554	0.190
Worker	11 (7.1%)	95.19 ± 21.06		
Farmer	10 (6.5%)	91.30 ± 16.87		
Civil servant or public institution staff	51 (33.1%)	98.84 ± 13.49		
Retiree	21 (13.6%)	93.86 ± 11.80		
Others	61 (39.6%)	93.37 ± 10.60		
Marital status			2.118	0.124
Single	26 (16.9%)	90.50 ± 12.02		
Married	121 (78.6%)	96.31 ± 13.43		
Divorced or widowed	7 (4.5%)	94.43 ± 10.78		
Living status			1.014	0.312
Living alone	31 (20.1%)	93.10 ± 13.03		
Not living alone	123 (79.9%)	95.79 ± 13.25		
Medical payment method			0.440	0.725
Self-paid	4 (2.6%)	90.25 ± 14.29		
Government-funded	3 (1.9%)	98.67 ± 13.20		
Medical insurance	137 (89.0%)	95.53 ± 13.30		
New Rural Cooperative Medical Scheme (NRCMS)	10 (6.5%)	92.30 ± 12.58		
Monthly household per capita income (Chinese Yuan, CNY)			1.979	0.120
<1000	2 (1.3%)	97.00 ± 8.49		
1000∼3000	10 (6.5%)	91.10 ± 8.36		
3001∼5000	39 (25.3%)	91.59 ± 13.92		
>5000	103 (66.9%)	97.00 ± 13.14		
Presence of similar cases among relatives or friends			−0.621	0.535
Yes	54 (35.1%)	96.15 ± 12.79		
No	100 (64.9%)	94.76 ± 13.47		
Length of hospital stay (day)			1.384	0.168
≥4	115 (74.7%)	94.39 ± 13.56		
<4	39 (25.3%)	97.77 ± 11.90		

College diploma*, junior college education in China.

### 3.2 RHDS, HADS, and SSRS scores of 154 postoperative thyroid cancer patients

Among postoperative thyroid cancer patients receiving ERAS-based perioperative management, the total RHDS score was 95.25 ± 13.21, with a mean item score of 7.94 ± 1.10. Among all RHDS dimensions, the highest mean item score was observed in expected support, while the lowest was in personal status. In terms of HADS assessment, the mean anxiety score was 8.12 ± 3.76, which was higher than the mean depression score of 6.41 ± 3.76. Regarding SSRS, its dimension scores were ranked from high to low as follows: subjective support, objective support, and utilization of social support. Detailed results are presented in Table 2.

**TABLE 2:** RHDS, HADS, and SSRS scores of postoperative thyroid cancer patients under ERAS protocol (*N* = 154, Mean ± SD).

Scale/dimension	No. of items	Score	Mean item score
RHDS	12	95.25 ± 13.21	7.94 ± 1.10
Personal status	3	22.80 ± 4.22	7.60 ± 1.41
Coping ability	5	39.40 ± 6.04	7.88 ± 1.21
Expected support	4	33.05 ± 6.20	8.26 ± 1.55
HADS	14	14.53 ± 6.81	1.04 ± 0.49
Anxiety	7	8.12 ± 3.76	1.16 ± 0.54
Depression	7	6.41 ± 3.76	0.92 ± 0.54
SSRS	10	41.24 ± 7.44	4.12 ± 0.74
Objective support	3	10.47 ± 3.21	3.49 ± 1.07
Subjective support	4	23.43 ± 4.64	5.86 ± 1.16
Utilization of social support	3	7.34 ± 1.89	2.45 ± 0.63

RHDS, Readiness for Hospital Discharge Scale; HADS, Hospital Anxiety and Depression Scale; SSRS, Social Support Rating Scale.

### 3.3 Correlation analysis of readiness for hospital discharge in postoperative thyroid cancer patients

Pearson correlation analysis showed that the total RHDS score of postoperative thyroid cancer patients was negatively correlated with the total HADS score (*r* = −0.777, *P* < 0.01), and positively correlated with the total SSRS score (*r* = 0.729, *P* < 0.01).

### 3.4 Multivariate analysis of factors associated with readiness for hospital discharge in postoperative thyroid cancer patients

The total RHDS score was taken as the dependent variable. Gender, total HADS score and total SSRS score, which showed statistically significant differences in previous analyses, were selected as candidate independent variables. In addition, variables considered clinically relevant potential predictors, such as age, education level, marital status, monthly household per capita income, length of hospital stay, living status, and the presence of similar cases among relatives or friends, were also entered into the initial regression model regardless of their statistical significance in univariate analyses. After collinearity testing and stepwise multiple regression screening, age, education level, marital status, monthly household per capita income, length of hospital stay, living status, and presence of similar cases among relatives or friends (*P* > 0.05), were excluded from the final model due to their weak explanatory power. The final multiple regression model retained only gender, the total HADS score, and the total SSRS score as variables significantly associated with RHDS scores (Table 3).

**TABLE 3:** Stepwise multiple regression analysis of factors associated with discharge readiness in postoperative thyroid cancer patients (*N* = 154).

Independent variable	*B*	*SE*	β	*t*	*P*	95% CI	VIF
Intercept	91.438	8.876	–	10.302	<0.001	(73.901, 108.975)	–
Total HADS score	−1.077	0.175	−0.555	−6.145	<0.001	(−1.424, −0.731)	3.298
Total SSRS score	0.472	0.160	0.266	2.942	0.004	(0.155, 0.789)	3.298

*F* = 126.297, *P* < 0.001, *R*^2^ = 0.626, Adjusted *R*^2^ = 0.621.

Stepwise multiple regression analysis indicated that total HADS score and total SSRS score were significantly associated with discharge readiness in postoperative thyroid cancer patients (Table 3). The final regression model explained 62.6% of the variance in RHDS scores (*R*^2^ = 0.626, adjusted *R*^2^ = 0.621).

Model diagnostics supported the adequacy of the final model. The scatter plot of standardized residuals against standardized predicted values showed a random distribution around zero, with no apparent curvature or funnel-shaped pattern, supporting the assumptions of linearity and homoscedasticity. The normal P–P plot indicated that the standardized residuals were approximately normally distributed. No evidence of problematic multicollinearity was observed (all VIF values < 5). Cook’s distance values were all below 1, suggesting that no influential observations had a substantial impact on the model.

## 4 Discussion

### 4.1 Status of readiness for hospital discharge, anxiety, depression, and social support among postoperative thyroid cancer patients under the ERAS protocol

This study showed that the total RHDS score of postoperative thyroid cancer patients was 95.25 ± 13.21. This finding was higher than that reported by Zeng et al. (2024), among patients undergoing lung cancer surgery but lower than that reported by Chen et al. (2026), among patients after pancreatic cancer resection. These differences suggest that discharge readiness cannot be explained by disease severity alone. It may be attributed to variations in disease characteristics, surgical procedures, healthcare settings, and treatment modalities among different patient populations.

Among the RHDS dimensions, the personal status dimension had the lowest mean item score, suggesting that patients had relatively limited confidence in their ability to cope with post-discharge conditions. Previous studies have shown that thyroid cancer patients managed under ERAS protocols can typically be discharged within 2–4 days after surgery (Chorath et al., 2022; Machado et al., 2024; Zheng and Zhang, 2022). However, the further reduction in hospital stay duration may limit the time available for patients and their families to prepare for discharge. For example, patients may have insufficient time to acquire knowledge and skills related to post-discharge care, including neck functional exercises and medication management, which may contribute to lower scores in the personal status dimension.

In contrast, the expected support dimension achieved the highest mean item score, which was consistent with the findings of Li et al. (2024). This result indicates that patients expected to receive substantial rehabilitation support and assistance after discharge. This expectation may be associated with the long-term care requirements of thyroid cancer patients after surgery. Following thyroid cancer surgery, patients often require lifelong thyroid hormone replacement therapy, regular thyroid function monitoring, and medication dose adjustments. In addition, regular cervical ultrasound follow-up is required, and some patients may experience short-term postoperative symptoms, such as neck stiffness, hoarseness, and swallowing discomfort. These ongoing healthcare needs may increase patients’ reliance on family members and social resources, resulting in a stronger expectation for support and assistance during the recovery process.

Regarding psychological status, the mean anxiety score on the HADS was (8.12 ± 3.76), which was higher than the mean depression score (6.41 ± 3.76), suggesting that postoperative thyroid cancer patients were more likely to experience anxiety than depression. This finding may be attributed to the unique characteristics of thyroid cancer. Although thyroid cancer is generally regarded as an indolent malignancy with a favorable prognosis and a high surgical cure rate, patients may still experience considerable concern regarding disease recurrence, metastasis, and long-term prognosis. In addition, many patients worry about whether surgery will affect their future work, daily activities, and overall quality of life.

Previous studies have suggested that negative psychological responses, such as anxiety and depression, may occur throughout the entire disease trajectory of thyroid cancer and are closely associated with patients’prognosis and clinical outcomes (Dionisi-Vici et al., 2021; Hamurcu et al., 2026; Kuang et al., 2025). Furthermore, postoperative thyroid cancer patients usually require long-term thyroid hormone replacement therapy. Concerns regarding medication adherence, dose adjustment, potential adverse effects, and lifelong treatment may further contribute to psychological distress.

These findings highlight the importance of incorporating psychological assessment and intervention into perioperative ERAS management. Healthcare professionals should pay close attention to patients’ emotional well-being, proactively communicate with patients to identify their concerns, and provide timely psychological support. In addition to strengthening perioperative health education under the ERAS protocol, individualized guidance on medication management, dietary recommendations, and postoperative self-care should also be provided. Improving patients’ understanding of thyroid cancer and its treatment may help alleviate anxiety, enhance self-management ability, and ultimately increase patients’ confidence in returning to normal daily life and work following surgery.

Regarding social support, the total SSRS score of postoperative thyroid cancer patients was 41.24 ± 7.44, indicating a moderate level of social support. Among the three dimensions of the SSRS, the highest mean item score was observed for subjective support (5.86 ± 1.16), followed by objective support (3.49 ± 1.07), while the lowest score was observed for utilization of social support (2.45 ± 0.63).

These findings suggest that, in daily life, thyroid cancer patients may receive emotional and psychological support from family members and close social networks more easily than material or practical support, which may be influenced by objective factors such as household economic conditions and available social resources. In addition, the relatively low score for the utilization of social support dimension indicates that patients may have limited ability to effectively access and utilize available support resources.

Therefore, during hospitalization, healthcare professionals should encourage patients to actively express their needs for assistance and provide guidance on how to effectively utilize available social support resources. Enhancing patients’ ability to seek and obtain support may help them better cope with illness-related challenges and facilitate postoperative recovery.

### 4.2 Factors associated with readiness for hospital discharge

Although male patients had significantly higher RHDS scores than female patients, consistent with the findings of Li et al. (2026), gender was not retained in the final multivariable regression model, and the observed gender difference should be interpreted cautiously. Further studies are warranted to clarify the mechanisms underlying gender differences in readiness for hospital discharge.

The stepwise multiple regression analysis in this study demonstrated that the total HADS and SSRS scores were significantly associated with readiness for hospital discharge among postoperative thyroid cancer patients. Previous studies have shown that psychological status is an important factor associated with patients’ readiness for hospital discharge (Presciutti et al., 2024; Wang et al., 2024). Patients with more severe anxiety and depressive symptoms tended to have lower levels of discharge readiness. However, given the cross-sectional design precludes determination of the temporal relationship between psychological distress and discharge readiness. Although higher HADS scores were associated with lower RHDS scores in the present study, reverse causality cannot be excluded: insufficient readiness for hospital discharge may also contribute to or exacerbate psychological distress. Patients who are inadequately prepared for discharge may experience anxiety and concerns regarding self-management after returning home, which could in turn increase psychological distress. Clinical practice should integrate routine assessment of both domains as part of discharge preparation. Healthcare professionals may use validated tools, such as the RHDS and HADS to identify patients with insufficient discharge readiness or heightened psychological distress at an early stage. For patients screened to be at risk, individualized discharge planning, intensified self-management education, and clear written discharge instructions may help address unmet support needs. Furthermore, appropriate referral for psychological support alongside structured post-discharge follow-up may also promote a seamless transition from hospital to home and improve the quality of transitional care. In addition, because both RHDS and HADS were self-reported questionnaires administered at the same time point, common-method bias cannot be completely excluded. Future studies could incorporate clinician-rated assessments or other independent data sources alongside patient-reported measures to reduce common-method bias and provide a more comprehensive evaluation of discharge readiness.

Furthermore, the present study demonstrated a positive association between social support and readiness for hospital discharge, consistent with the findings reported by Huang et al. (2022). Previous studies have indicated that sufficient social support and nursing assistance can alleviate patients’ adaptation burden after discharge and enhance their confidence in coping with recovery, thereby contributing to higher readiness for hospital discharge (Wang Y. et al., 2026; Wang D. et al., 2026). It should be noted that the Expected Support dimension of the RHDS assesses patients’ anticipated availability of support after discharge, whereas the SSRS evaluates perceived social support from objective support, subjective support, and support utilization. Although these instruments measure distinct constructs in this study, they both capture support-related aspects of patients’ post-discharge experience. Therefore, part of the observed association may reflect shared conceptual elements between the measures rather than entirely independent constructs. Nevertheless, the significant association suggests that patients who perceive greater support resources also tend to report greater readiness for the transition from hospital to home.

Therefore, healthcare professionals should consider both psychological and social factors when implementing discharge preparation interventions. In addition to providing routine discharge education and medical guidance, nurses should assess patients’ psychological status, address their concerns regarding postoperative recovery, and provide individualized support, such as guidance on neck functional exercises, medication management, and disease-related questions. Meanwhile, family involvement should be encouraged to provide emotional, practical, and financial support when appropriate. Structured caregiver education programs may further enhance caregivers’ ability to participate in the rehabilitation process. Promoting patients’ active utilization of available social support resources may help establish effective patient–caregiver partnerships, facilitate postoperative recovery, help support discharge preparation, and optimize the utilization of healthcare resources.

Meanwhile, although variables such as living status, length of hospital stay, and presence of similar case among relatives or friends were not significantly associated with discharge readiness, yet certain clinical trends were observed. Patients not living alone tended to slightly higher discharge readiness scores compared to those living alone, patients hospitalized for less than 4 days demonstrated slightly higher discharge readiness scores, and patients with similar case among relatives or friends scored slightly higher on RHDS. Considering the modest sample size of this study, these clinical trends should be interpreted cautiously and require further validation through large-sample studies to confirm whether these variables are associated with discharge readiness in the future, aiming to provide more evidence-based guidance for clinical practice.

### 4.3 Clinical implications for discharge preparation in postoperative thyroid cancer patients within ERAS-based perioperative care

Within ERAS-based perioperative care, discharge preparation should be initiated throughout the perioperative period rather than being concentrated on the day of discharge. The 2–4– day hospital stay under the ERAS pathways may compress the time available for patients and their families to prepare for discharge. Although this possibility was not directly examined in the present study, it may partially explain why the personal status dimension of RHDS scored the lowest in this study. Some patients may have insufficient time to adapt to their postoperative physical condition or to fully master recovery-related knowledge and skills, which may negatively affect their judgment of their own coping ability after discharge. This potential clinical implication requires controlled studies to examine in the future.

Our study has several limitations. First, it was a cross-sectional design and it can’t permit determination of temporal relationships among psychological distress, social support, and discharge readiness. Second, all participants received ERAS-based perioperative management, and no non-ERAS comparison group was included. Therefore, the present study cannot evaluate the effectiveness of ERAS itself. Third, this study employed a single-center convenience sampling design, which may have introduced selection bias and limited the representativeness of the sample. Restricted by the clinical characteristics of the participating institution, the sample may not fully represent the patient population in other healthcare settings implementing ERAS protocols. Therefore, the findings cannot yet be generalized to all institutions. In the future, we should expand the research scope and conduct multi-center studies to improve the generalizability and representativeness of the results. Fourth, although demographic and psychosocial variables were included in the analysis, other potentially relevant factors were not assessed. These included surgical characteristics (e.g., the extent of thyroidectomy and whether lymph node dissection was performed), postoperative symptom burden (e.g., hoarseness, dysphagia, and neck discomfort), variations in the quality of discharge education provided by different educators, family caregiving capacity, and thyroid hormone levels. These unmeasured variables may have contributed to residual confounding and should be considered in future studies. Fifth, all variables were assessed using self-reported questionnaires, which may introduce reporting bias and common-method bias. Future research should also consider incorporating assessments from healthcare professionals to complement patient-reported measures and provide a more comprehensive evaluation of discharge readiness. Finally, because both the RHDS and SSRS include support-related constructs, the observed association between the two measures should be interpreted with caution given their potential conceptual overlap.

## 5 Conclusion

In conclusion, among postoperative thyroid cancer patients receiving ERAS-based perioperative management, greater psychological distress was associated with lower readiness for hospital discharge, whereas higher levels of social support were associated with greater readiness for hospital discharge. These findings highlight the importance of incorporating psychological assessment, social support evaluation, and individualized discharge preparation into perioperative care. Future prospective studies with appropriate comparison groups are needed to further investigate factors associated with discharge readiness across different perioperative care pathways.

## Data Availability

The raw data supporting the conclusions of this article will be made available by the authors, without undue reservation.
